# Association of Householder Smoking With Poverty and the Mediating Effect of NCDs in Relatively Underdeveloped Regions in China

**DOI:** 10.3389/fpubh.2022.858761

**Published:** 2022-05-19

**Authors:** Huimin Yang, Bowen Chen, Aili Guo, Jiarui Song, Xi Cheng, Chenggang Jin

**Affiliations:** ^1^Department of Child Health Development, Capital Institute of Pediatrics, Beijing, China; ^2^Community Health Association of China, Beijing, China; ^3^School of Social Development and Public Policy, Beijing Normal University, Beijing, China; ^4^Research Center for Health and Social Policy, Beijing Normal University, Zhuhai, China

**Keywords:** cigarette smoking, poverty, noncommunicable diseases (NCDs), extended probit regression, generalized structural equation model

## Abstract

**Background:**

Studies have not provided clear enough evidence on the direct association between cigarette smoking and poverty. This study aims to assess the association of householder smoking with near-poverty households, and the potential mediating effect of NCDs.

**Methods:**

A cross-sectional survey was conducted from November 2019 to October 2020 in relatively underdeveloped regions in China. In total, 2,409 households were investigated in areas under the jurisdiction of 24 primary health care (PHC) institutions of eight provinces. Pearson's χ^2^-test was performed, and multivariable logistic regression and extended probit regression models were fitted to examine the association between householder smoking and near-poverty households. Moreover, generalized structural equation modeling was used to explore the mediating effect of NCDs.

**Results:**

After adjusting for all other potential confounding factors, compared with households headed by never-smokers, households headed by smokers exhibited significantly elevated risks of being near poverty, with an odds ratio of 2.01 (95% CI: 0.48–0.91). We also found that living in rural areas and having a low education level both had a negative effect on being near poverty. Additionally, NCDs had a significantly positive mediating effect, with a 31.57% effect of householder smoking on near-poverty status mediated by NCDs; the indirect effect was estimated to be 0.17 (95% CI: 0.04–0.31).

**Conclusions:**

Householder smoking significantly elevated the risk of the household being near poverty, and suffering NCDs had a positive mediating effect.

## Introduction

Cigarette smoking is a major public health problem and remains the leading preventable cause of death and disability in China and other countries, killing more than 8 million people a year around the world (1). In 2020, it was estimated that there were approximately 1.3 billion tobacco users worldwide, with the vast majority living in low- and middle-income countries or in more disadvantaged socioeconomic groups (1, 2). China is the world's largest producer and consumer of tobacco, accounting for more than 44% of the world's total cigarette consumption (3). It is estimated that one million people die of tobacco-related diseases in China every year, with the majority of those individuals in their productive years. Unfortunately, this number is estimated to exceed three million per year by the end of 2050 if the smoking epidemic is not controlled (4, 5).

Additionally, even though the global percentage of people living in extreme poverty declined from 36% in 1990 to 10% in 2015, more than 700 million people now live in extreme poverty (6). “End poverty in all its forms everywhere” is the first goal of United Nations Sustainable Development. Studies have found that noncommunicable diseases (NCDs), associated with mortality and prolonged disability, have negative impacts at the individual, community, and societal levels (7) and could expose individuals to poverty through lost productivity, unemployment and long-term medical expenses (8, 9). Meanwhile, studies have also found that cigarette smoking is one of the main risk factors driving the growing epidemic of NCDs, including cancers and cardiovascular and respiratory diseases (10, 11). However, early studies have not provided clear enough evidence on the direct association between cigarette smoking and poverty. Evidence is accordingly required to clarify the association of cigarette smoking with poverty and its underlying mechanisms, underpinning the rationale for integrating tobacco control policies with poverty alleviation strategies.

At the macro level, multiple studies have demonstrated that tobacco use imposes an economic burden through a reduction in productivity and an increase in the cost of medical treatment (12). For example, Pearce et al. (13) reported that in 2012, tobacco use contributed to an estimated USD 7.9 billion, USD 402 million, and USD 138 million in lost productivity in China, Brazil, and South Africa, respectively. Cigarette smoking accounted for approximately USD 289-332.5 billion in medical expenses over the period 1964–2014 in the United States (14), while in Indonesia, tobacco-related treatment costs were estimated at nearly USD 2.2 billion (15). The Directorate General for Health and Consumers study (16) reported that the estimated costs attributed to cigarette smoking in the European Union amounted to approximately USD 714.9 billion in 2009, and a global estimated economic burden of smoking was approximately USD 1.44 trillion in 2012 (17).

At the micro level, there have been few studies about cigarette smoking and poverty. Liu et al. (18) estimated cigarette smoking's impact on poverty through excessive medical spending and direct spending on cigarettes. Wei et al. (19) conducted a population-based study and demonstrated that smoking can significantly reduce the income of Chinese urban residents, resulting in immense negative impacts on society. In the traditional Chinese family structure, the householder is often male and males have much higher smoking rates than females (5). Moreover, householders often shoulder a heavy burden and play a central role in households' economic status. However, to our knowledge, no study has assessed the association between householder smoking and poverty and its underlying mechanisms. To fill this research gap, we conducted a cross-sectional study and assessed the association of householder smoking with near-poverty households and the mediating effect of NCDs in relatively underdeveloped regions in China.

## Methods

### Study Design and Participants

We conducted a cross-sectional questionnaire-based survey in relatively underdeveloped regions in China from November 2019 to October 2020, and the participants were enrolled using a stratified multistage sampling method. According to per capita GDP levels, we first chose 12 cities/counties that had relatively lower per capita GDP in eight province-level regions in China (North China: Fuping and Pingshan in Hebei Province; Central China: Nanyang in Henan Province, Macheng and Qichun in Hubei Province; Eastern China: Linqing and Wuli in Shandong Province; Southwest China: Luzhou in Sichuan Province, Kaili in Guizhou Province; Northwest China: Yulin in Shanxi Province; Northeast China: Harbin and Wuchang in Heilongjiang Province). Then, in each city/county, two primary health care (PHC) institutions were randomly selected as our investigation units, for a total of 24 PHC institutions.

Next, with the coordination of local health professionals in PHC institutions, approximately 100 households were selected in areas under the jurisdiction of each PHC institution. The participants were not sampled randomly but were directly selected based on the following: (1) living in local communities for at least 6 months; (2) willing to participate in this study; (3) the householder was aged over 40; (4) near-poverty households and nonpoor households were included; and (5) smokers and never-smokers were included.

### Data Collection and Measures

The selected householders had a face-to-face interview with the local health professionals of the PHC institutions, and prior to the interview, investigators received professional training to ensure data collection quality. A validated interviewer-administered questionnaire was used to obtain information about the household's basic situation (household income, size of household and residence location), demographic characteristics of the householder (sex, age, education level, marital status, and income level), and the householder's smoking status, whether the householder suffered from NCDs or not and so on. All measures were self-reported in the study.

### Poverty Status

In 2019, the Chinese government defined the poverty as yearly per capita income <3,747 Yuan (or US $1.49 per day). In our study, we further defined near-poverty as 200% of the poverty level definition (7,494 Yuan) and categorized household poverty status as near-poverty (yearly per capita income <7,494 Yuan) or nonpoor (yearly per capita income of 7,494 Yuan or above).

### Smoking Status

Smoking status was categorized as smoker (including current smoker and former smoker) or never-smoker. Participants were asked: “Have you smoked in the last month?” When the respondent answered “yes”, the individual was categorized as a “current smoker”; if the respondent answered “no”, he or she was then asked, “have you ever smoked?” If the respondent answered “yes”, the individual was considered to be a “former smoker”; if the answers to both questions were “no”, the respondent was considered a never-smoker.

### Noncommunicable Diseases (NCDs)

Noncommunicable disease status were determined by the following question: “Have you ever been diagnosed with noncommunicable diseases, such as hypertension, diabetes, COPD, CHD, strokes, cancer and so on?” Respondents who replied “yes” were categorized as having NCDs.

### Respondent Characteristics

We controlled for an array of demographic and socioeconomic statuses that have been previously shown to be associated with cigarette smoking and poverty. The basic household situation included the household's residential location (rural or urban) and household size (number of household members). Demographic characteristics of the householder included the following: sex (male or female); age group (40–50 years, 50–60 years, 60–70 years, ≥70 years); educational level (illiterate, primary, junior high, senior high/vocational, college and above), marital status (single, married, divorced/widowed) and alcohol use (yes or no). We also asked, “Does raising cigarette prices influence tobacco use?” (yes or no). All covariates were categorical variables.

### Statistical Analysis

The database was established using Epidata 3.1 and transferred into Stata/MP 16.0 software for analysis. Categorical (nominal/ordinal) variables were described using frequencies and percentages. Pearson's χ^2^-test was performed to assess differences in the characteristics between near-poverty and nonpoor households for the categorical variables. Both univariate and multivariate logistic regression models were used to determine the association between householder smoking and near-poverty households. The covariates were adjusted in the multivariable logistic regression model. Moreover, to accommodate endogenous covariates, we fitted an extended probit regression model (20) using the response to “Does raising cigarette prices influence tobacco use?” as the instrumental variable of smoking status to examine the association. In addition, we used generalized structural equation modeling to evaluate the mediating effect of NCDs.

## Results

### Demographic Characteristics

The study included 1,595 households from rural areas and 814 households from urban areas. Of the 2,409 households, 430 (17.85%) households were near-poverty, and 1,979 (82.15%) households were nonpoor. A total of 1,183 (49.11%) householders were never-smokers, while 1,226 (50.89%) householders were smokers. The ages of the householders ranged from 40 to 80 years, with an average age of 55.52 ± 10.42 years. Table 1 compares the basic household situations and the householders' demographic characteristics according to poverty status. Near-poverty households were more likely to be in rural areas and to have only one member in the household. For householders from near-poverty households, the smoking rate was higher than that in householders from nonpoor households (60.00 vs. 48.91%, *P* < 0.001). NCDs were reported to be more prevalent among near-poverty householders (51.16%) than nonpoor householders (30.32%). Furthermore, near-poverty householders tended to be older, to be single or divorced/widowed, and to have a lower education level. In terms of householder sex, there were no considerable differences between the two categories of households (84.19 vs. 84.13% male).

**Table 1:** The sociodemographic characteristics of near-poverty and nonpoor households.

**Characteristics**	**Near-poverty N(%)**	**Nonpoor N(%)**	**χ^2^**	***P*** **value**
Smoking status			17.37	<0.001
Never-smoker	172 (40.00)	1,011 (51.09)		
Smoker	258 (60.00)	968 (48.91)		
Residence location			132.41	<0.001
Urban	43 (10.00)	771 (38.96)		
Rural	387 (90.00)	1,208 (61.04)		
Household size^*^			97.37	<0.001
1	101 (23.60)	151 (7.68)		
2	118 (27.57)	665 (33.84)		
3–4	128 (29.91)	777 (39.54)		
≥5	81 (18.93)	372 (18.93)		
Sex			<0.001	0.978
Male	362 (84.19)	1,665 (84.13)		
Female	68 (15.81)	314 (15.87)		
Age group			91.89	<0.001
40–50 years	83 (19.30)	749 (37.85)		
50–60 years	129 (30.00)	642 (32.44)		
60–70 years	124 (28.84)	394 (19.91)		
≥70 years	94 (21.86)	194 (9.80)		
Education level^*^			430.36	<0.001
Illiterate	129 (30.07)	86 (4.36)		
Primary	165 (38.46)	412 (20.90)		
Junior high	109 (25.41)	741 (37.60)		
Senior high/vocational	24 (5.59)	424 (21.51)		
College and above	2 (0.47)	308 (15.63)		
Marital status^*^			226.06	<0.001
Single	62 (14.45)	25 (1.26)		
Married	280 (65.27)	1,759 (88.93)		
Divorced/Widowed	87 (20.28)	194 (9.81)		
NCDs			54.28	<0.001
Yes	210 (51.16)	600 (30.32)		
No	220 (48.84)	1,379 (69.68)		

* *There were missing data, and the variables of household size, education level and marital status had 16, 9 and 2 missing data points, respectively*.

### Association of Householder Smoking With Near-Poverty Household

Table 2 shows the association of householder smoking with near-poverty status among households in relatively underdeveloped regions of China. The results of Models 1–3 all suggest that householder smoking significantly increased the risk of households being near poverty (*P* < 0.05). As shown in Models 1–2, compared to households headed by never-smokers, households headed by smokers had an approximately 50% increase in the probability of household being near poverty, with odds ratios of 1.57 (95% CI: 1.27–1.94) and 1.49 (95% CI: 1.14–1.95), respectively. In Model 3, we found that the correlation between the errors of our two equations was significantly negative, so the unobservable factors that increased the householder smoking rates decreased the probability of households being near poverty. The results of Model 3 revealed that households headed by smokers had a two times higher risk of being near poverty than households headed by never-smokers, with an odds ratio of 2.01 (95% CI: 0.48–0.91).

**Table 2:** Unadjusted and adjusted odds ratios of the association of near-poverty status with householder smoking.

**Variable**	**Model 1 OR (95% CI)**	**Model 2 OR (95% CI)**	**Model 3 OR (95% CI)**
**Poverty status**			
Smoking status (Ref.: Never-smoker)			
Smoker	1.57 (1.27–1.94) ^***^	1.49 (1.14 to 1.95) ^**^	2.01 (0.48 to 0.91) ^***^
Residence location (Ref.: Rural)			
Urban		0.50 (0.33 to 0.74) ^***^	0.70 (−0.56 to −0.15) ^***^
Age group (Ref.: 40–50 years)			
50–60 years		1.18 (0.84 to 1.65)	1.11 (−0.08 to 0.28)
60–70 years		1.17 (0.81 to 1.69)	1.10 (−0.10 to 0.30)
≥70 years		1.18 (0.76 to 1.83)	1.124 (−0.12 to 0.36)
Sex (Ref.: male)			
Female		0.87 (0.56 to 1.36)	1.18 (−0.08 to 0.41)
Education level (Ref.: illiterate)			
Primary		0.33 (0.23 to 0.49) ^***^	0.52 (−0.87 to −0.43) ^***^
Junior high		0.13 (0.08 to 0.19) ^***^	0.31 (−1.42 to −0.94) ^***^
Senior high/vocational		0.06 (0.03 to 0.11) ^***^	0.21 (−1.86 to −1.25) ^***^
College and above		0.01 (0.01 to 0.05) ^***^	0.12 (−2.73 to −1.57) ^***^
Marital status (Ref.: single)			
Married		0.10 (0.05 to 0.18) ^***^	0.27 (−1.65 to −1.00.) ^***^
Divorced/widowed		0.22 (0.11 to 0.44) ^***^	0.42 (−1.25 to −0.51) ^***^
**Smoking status**			
Influence or not (Ref.: NO)			
Yes			18.47 (2.43 to 3.41) ^***^
Sex (Ref.: male)			
Female			0.33 (−1.28 to −0.92) ^***^
Corr (e. smoking status, e. poverty status)			0.67 (−0.53 to −0.24) ^***^

*Model 1 is unadjusted. Model 2 is adjusted for residence location, education level, marital status and provinces. Based on Model 2, Model 3 was fitted using the response to the question about higher cigarette prices influencing tobacco use as the instrumental variable, with sex was also adjusted in the auxiliary model*.

*The estimated results for the variable province are not shown in the table*.

** *p < 0.01;*

*** *p < 0.001*.

Table 3 presents the average treatment effect (ATE) of householder smoking on near-poverty status and the average potential-outcome means (PO means) of the two smoking status based on Model 3. When all householders were never-smokers, we estimated that the average probability of households being near poverty was 12.14% (95% CI: 0.10–0.14), which would rise to 25.23% (95% CI: 0.22–0.28) when all householders were smokers. The average probability of households being near poverty increased by 12.99% (95% CI: 0.09–0.17) when all householders were smokers vs. when all householders were never-smokers.

**Table 3:** Estimates of ATE of householder smoking on being near poverty and PO means of the two smoking status.

	**Margin**	**Unconditional standard errors**	**z**	***P*** **value**	**95% CI**
**PO mean**					
Smoking status					
Never-smoker	0.1223766	0.0097019	12.61	<0.001	0.10–0.14
Smoker	0.2522712	0.0166563	15.15	<0.001	0.22–0.28
**ATE**					
Smoking status					
(Smoker vs. never-smoker)	0.1298946	0.0214413	6.06	<0.001	0.09–0.17

**Figure 1:**
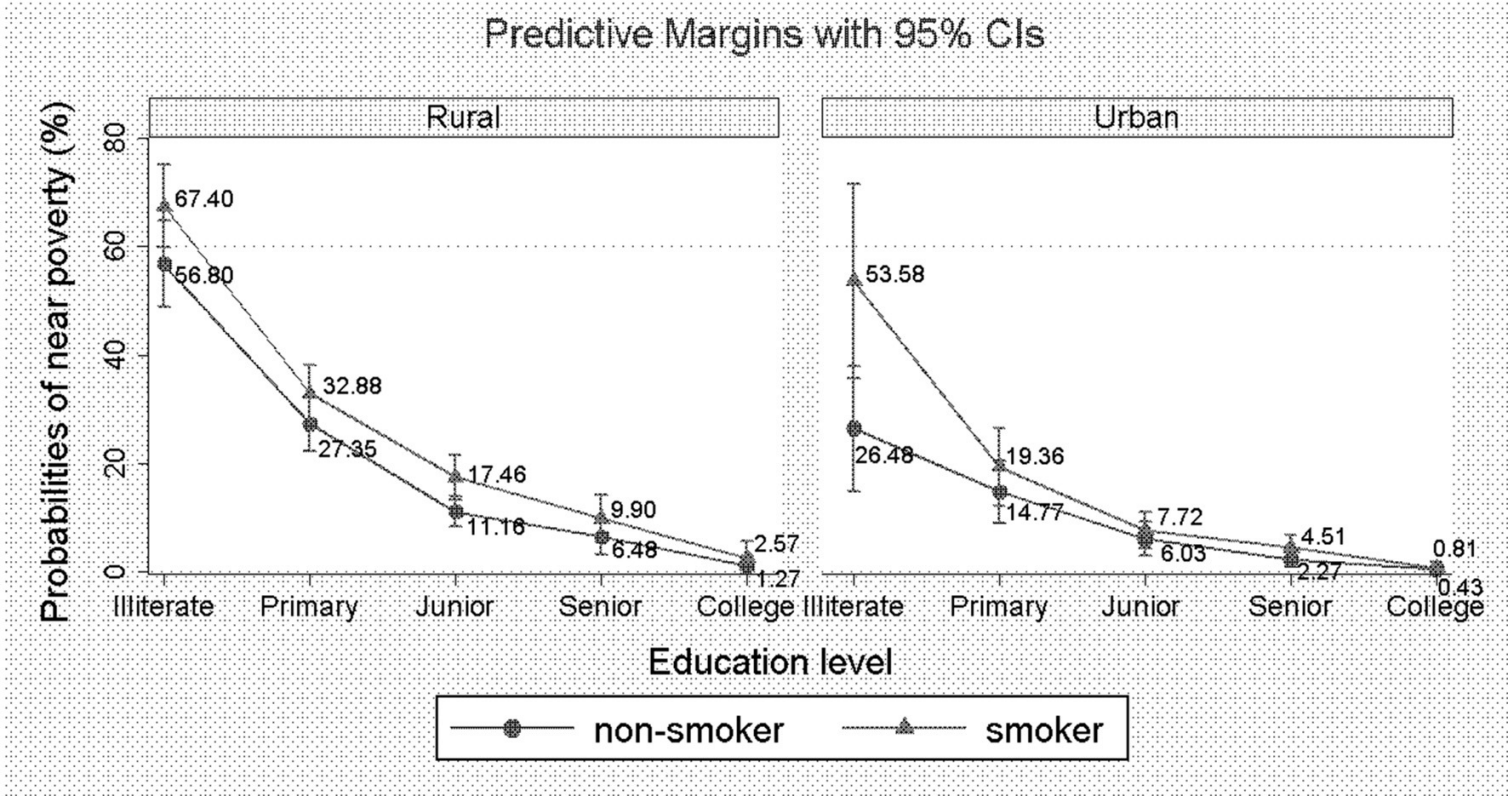
Predictive probabilities of being near poverty across different education levels and smoking status.

Moreover, households in rural areas had higher odds of being near poverty than households in urban areas (OR = 0.70, 95% CI: −0.56 to −0.15). Additionally, we also found that the higher the education level of the householder was, the lower the predictive probability of the household being near poverty in both rural and urban areas. However, regarding different education levels, households headed by smokers all had higher probabilities of being near poverty than households headed by never-smokers (Figure 1).

### Mediating Effect of NCDs Between Householder Smoking and Near-Poverty Status

**Figure 2:**
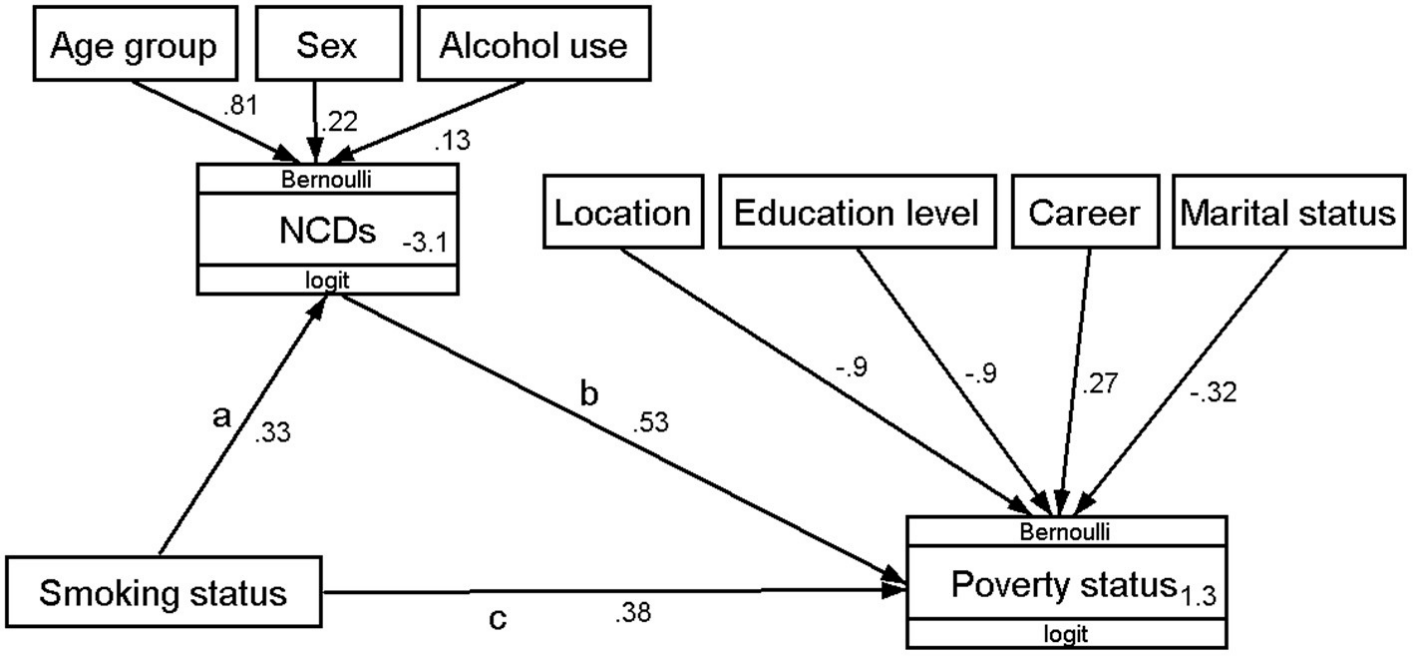
Path diagram of generalized structural equation model estimation of the mediating effect of NCD's between householder smoking and near-poverty status. Path c is the direct effect before taking into account the effect of NCDs. Path a and b make up the mediating pathway, with the mediating effect usually being described in the literature as the product of co-efficient (ab). Age group, sex, and alcohol use were adjusted as confounding variables of NCDs. Residence location, education level, career, and marital status were adjusted as the confounding variables of being near poverty.

Figure 2 presents the path diagram of the generalized structural equation model estimation used to evaluate the mediating effect of NCDs between householder smoking and near-poverty status. As shown in Table 4, smokers had an increased risk of suffered from NCDs compared with never-smokers, and the direct effect of smoking on NCDs was estimated to be 0.33 (95% CI: 0.13–0.53). Householders who had NCDs had an increased probability of being near poverty, and the direct effect was estimated to be 0.53 (95% CI: 0.28–0.77). There was a significant positive indirect effect of householder smoking on households being near-poverty status (coefficient: 0.17; 95% CI: 0.04–0.31). In addition, we also observed that the direct and total effects of householder smoking on near-poverty status were estimated to be 0.38 (95% CI: 0.14–0.62) and 0.55 (95% CI: 0.28–0.82), respectively, which were both significant. Overall, we estimated that 31.57% of the total effect was mediated by NCDs.

**Table 4:** Generalized structural equation model estimation results of the mediating effect of NCDs between householder smoking and near-poverty status.

	**Coefficient**	**Standard errors**	**z**	***P*** **value**	**95% CI**
**Direct effects**					
Poverty status					
NCDs	0.5276675	0.1240981	4.25	<0.001	0.28–0.77
Smoking status	0.3779753	0.1235824	3.06	0.002	0.14–0.62
NCDs					
Smoking status	0.3305322	0.1023171	3.23	0.001	0.13–0.53
**Indirect effects**					
Poverty status					
Smoking status	0.1744111	0.0678039	2.57	0.010	0.04–0.31
**Total effects**					
Poverty status					
Smoking status	0.5523864	0.1387412	3.98	<0.001	0.28–0.82

## Discussion

In this study, we observed a strong association between householder smoking and household being near-poverty status that persisted after adjusting for basic household situation and sociodemographic factors. In line with previous studies (19, 21), our results indicated that householder smoking elevated the probability of households being near poverty after adjustment for relevant covariates and potential confounds, which contributed evidence on the direct association between householder smoking and near-poverty status at the micro level.

Moreover, our study revealed a higher impact of smoking on poverty than in a previous study (19). Possible reasons are as follows. First, near-poverty status was used as the income criterion in this study, which was defined as 200% of the poverty level definition. Second, the study was conducted in relatively underdeveloped regions in China, particularly rural regions where residents have higher rates of smoking and poverty than residents of urban areas (22). Third, participants interviewed in this study were aged over 40, and studies have shown that adverse effects of smoking are cumulative and delayed, which would be gradually shown after smokers become middle-aged (14, 18). Meanwhile, we also found that the effect of householder smoking on household near-poverty status was more significant in households with householders aged over 50 than in households with householders under 50 years old.

The purchasing of cigarettes imposes a financial burden on low-income smokers and their families, but tobacco-related diseases, such as NCDs, exact an even greater cost and plunge those already on the margins into poverty. This study revealed that NCDs had a significantly positive mediating effect between householder smoking and household being near-poverty status, which offered evidence to consider the underlying mechanisms of the observed association. Potential explanations include the toxic effects of smoking on the human body, including but not limited to endothelial dysfunction, inflammation, and cancer, leading to a higher chance of developing NCDs (23, 24). Furthermore, NCDs result in reduced workforce participation and productivity, which has a direct bearing on household income (12, 19). Additionally, it has been suggested that NCDs significantly increase outpatient and hospitalization rates and medical expenditures, diverting household funds from basic necessities such as food, education and health care and further increasing the risk of poverty (25, 26). However, the causality of householder smoking and poverty is complex, and further studies should be carried out to provide more evidence.

Our study indicated that cigarette smoking has a strong association with near-poverty status, so from a policy perspective, controlling tobacco use and reducing smoking prevalence are not only important public health issues but are also closely related to poverty alleviation. As the most populated developing country in the world, China has made remarkable progress in poverty alleviation; however, problems of “sickness poor” and “poverty due to illness” remain the biggest barriers to consolidating achievements in poverty alleviation. Therefore, it is recommended to integrate tobacco control strategies with national poverty alleviation policies, which will be helpful in overcoming economic and political obstacles in the implementation of existing tobacco control measures. Additionally, we also found that NCDs mediated the association between householder smoking and near-poverty status, so it is recommended to strengthen health education and expand the coverage of medical insurance, which will be useful to lighten near-poverty households' financial burden and solve near-poverty households' inclination toward poverty because of illness. Furthermore, worldwide evidence has shown that raising tobacco taxes had the single greatest impact on the reduction of tobacco consumption (27, 28), and studies have demonstrated that low-income smokers were more price-sensitive and could reap greater health gains from increased taxes and higher prices of cigarettes (29, 30). It is also recommended to gradually increase the tobacco tax rate and prices to curb the tobacco epidemic.

We also noted that a low educational level of the householder was significantly associated with a higher probability of being near poverty. Previous studies have shown that less-educated individuals and individuals with lower socioeconomic status were more likely to be smokers (31, 32). In addition, studies have demonstrated that individuals with lower socioeconomic status also benefitted less from smoking prevention information (33, 34). Therefore, interventions aimed at preventing addiction to smoking among lower-educated individuals and individuals with lower socioeconomic status are recommended, including improving the educational and cognitive levels of individuals in relatively underdeveloped regions and promoting publicity campaigns about the hazards of smoking. In addition, due to lower awareness about the hazards of smoking and the benefits of smoking cessation aids and smoking cessation programs, individuals with lower socioeconomic status were less likely to quit smoking (35, 36). Hence, in future smoking cessation interventions, smokers with lower education and socioeconomic status should be given more attention.

The strengths of our study include the nationally representative survey. A broad range of data from relatively underdeveloped regions in China was collected, supplementing the empirical research conclusions on the relationship between householder smoking and household being near-poverty status. Moreover, in the study of the effects of householder smoking on near-poverty status, data endogeneity was unavoidable, and an extended probit regression model with the instrumental variable was used to overcome endogeneity problems. In addition, we used generalized structural equation modeling to evaluate the mediating effect of NCDs, providing evidence to consider the underlying mechanisms of the association between householder smoking and near-poverty households. However, there are also several limitations in this study. First, this study is a retrospective self-reported survey, and recall bias may be inevitable; however, prior studies have found that self-reported smoking status and health conditions had reasonable validity (37, 38). Second, the analysis was based on cross-sectional analyses, which limited our ability to make causal inferences about the association of householder smoking with being near poverty, and longitudinal association should be explored in future studies. Third, although we attempted to adjust several potential confounders, the analysis of the relationship of smoking and NCDs might miss potential important confounding variables due to lack of information such as diet and physical activity information.

## Conclusions

In conclusion, householder smoking significantly elevated the risk of households being near poverty, and suffering NCDs had a positive mediating effect between householder smoking and near-poverty status. We also found that a lower educational level and living in rural areas were significantly associated with a higher probability of being near poverty.

### What Is Already Known on This Subject?

- At the macro level, tobacco use imposes an economic burden through a reduction in productivity and an increase in the cost of medical treatment.
- At the micro level, few studies estimate the impact of cigarette smoking on poverty by reducing the income of Chinese urban residents and excessive medical spending.

### What This Study Adds?

- At the micro level, householder smoking significantly elevated the risk of households being near poverty.
- Suffering from NCDs has a positive mediating effect on the relationship between householder smoking and households being near poverty.

## Data Availability Statement

The datasets presented in this article are not readily available because data involves personal privacy issues. Requests to access the datasets should be directed to HY, yanghuimin85@126.com.

## Ethics Statement

This study was approved by the Ethics Committee of the Capital Institute of Pediatrics, Beijing (ID: SHERLL2020017). The patients/participants provided their written informed consent to participate in this study.

## Author Contributions

CJ, BC, and HY conceptualized the study. The questionnaire was developed by CJ, BC, and AG and refined with input from HY. Data collection in the field was coordinated by BC and AG and supervised by HY and JS. HY, JS, and XC contributed substantially to data analyses and interpretation of the results under the guidance of CJ. HY drafted the manuscript. CJ and BC revised the manuscript critically for intellectual content. All authors approved the final version of the manuscript.

## Funding

This work was supported by the Capital Institute of Pediatrics's Research Foundation (QN-2020-21) and the Capital's Funds for Health Improvement and Research (CFH 2020-3-1132).

## Conflict of Interest

The authors declare that the research was conducted in the absence of any commercial or financial relationships that could be construed as a potential conflict of interest.

## Publisher's Note

All claims expressed in this article are solely those of the authors and do not necessarily represent those of their affiliated organizations, or those of the publisher, the editors and the reviewers. Any product that may be evaluated in this article, or claim that may be made by its manufacturer, is not guaranteed or endorsed by the publisher.
